# Two years’ experience with denosumab for children with Osteogenesis imperfecta type VI

**DOI:** 10.1186/s13023-014-0145-1

**Published:** 2014-09-26

**Authors:** Heike Hoyer-Kuhn, Christian Netzer, Friederike Koerber, Eckhard Schoenau, Oliver Semler

**Affiliations:** Children’s Hospital, University of Cologne, Kerpenerstr. 62, D-50931 Cologne, Germany; Institute of Human Genetics, University of Cologne, Cologne, Germany; Institute for Radiology, University of Cologne, Cologne, Germany

**Keywords:** Osteogenesis imperfecta VI, SERPINF1, RANKL Antibody, Denosumab, Bone mineral density

## Abstract

**Background:**

Osteogenesis imperfecta (OI) is a hereditary disease causing reduced bone mass, increased fracture rate, long bone deformities and vertebral compressions. Additional non skeletal findings are caused by impaired collagen function and include hyperlaxity of joints and blue sclera. Most OI cases are caused by dominant mutations in *COL1A1/2* affecting bone formation. During the last years, recessive forms of OI have been identified, mostly affecting posttranslational modification of collagen. In 2011, mutations in *SERPINF1* were identified as the molecular cause of OI type VI, and thereby a novel pathophysiology of the disease was elucidated. The subgroup of patients with OI type VI are affected by an increased bone resorption, leading to the same symptoms as observed in patients with an impaired bone formation. Severely affected children are currently treated with intravenous bisphosphonates regardless of the underlying mutation and pathophysiology. Patients with OI type VI are known to have a poor response to such a bisphosphonate treatment.

**Method:**

Deciphering the genetic cause of OI type VI in our 4 patients (three children and one adolescent) led to an immediate translational approach in the form of a treatment with the monoclonal RANKL antibody Denosumab (1 mg/kg body weight every 12 weeks).

**Results:**

Short-term biochemical response to this treatment was reported previously. We now present the results after 2 years of treatment and demonstrate a long term benefit as well as an increase of bone mineral density, a normalization of vertebral shape, an increase of mobility, and a reduced fracture rate.

**Conclusion:**

This report presents the first two-year data of denosumab treatment in patients with Osteogenesis imperfecta type VI and in Osteogenesis imperfecta in general as an effective and apparently safe treatment option.

## Introduction

Osteogenesis imperfecta (OI) is a genetically heterogeneous hereditary disease characterized by reduced bone mass, increased fracture rate, vertebral collapses and deformities of long bones. Non-skeletal findings, e.g., dentinogenesis imperfecta, blue sclera, or hyperlaxity of ligaments, can be associated. Dominant mutations in *COL1A1* or *COL1A2* that lead to a quantitative or a qualitative defect in collagen type I are the molecular cause in the majority of patients [1]. Severely affected individuals are treated with intravenous bisphosphonates regardless of the underlying genetic cause [2,3].

Osteogenesis imperfecta type VI (OI VI) is autosomal-recessively inherited and displays an increased amount of non-mineralized osteoid and a poor response to bisphosphonate treatment [4,5]. Additional signs are the only discrete findings at birth and the late onset of fractures and deformities. OI VI is caused by mutations in *SERPINF1,* a gene which is coding for the pigment epithelium-derived factor (PEDF) [6,7]. In patients with bi-allelic truncating mutations in *SERPINF1,* PEDF is not detectable in the serum [8]. In-vitro and in-vivo models provided evidence that PEDF inhibits osteoclast differentiation and hence bone resorption *via* osteoprotegerin (OPG) and RANKL [9]. Receptor activator of NF-kB (RANK), the ligand RANKL, and the decoy receptor OPG are pivotal regulators of osteoclast differentiation and function. Denosumab is a monoclonal RANKL-blocking antibody which inhibits osteoclast formation and bone degradation and increases bone mass. It has been approved for the treatment of postmenopausal osteoporosis in 2010 and of giant cell tumors of the bone in 2013 [10,11].

We report the first 2-year results of four patients with genetically confirmed OI VI treated with denosumab. Understanding the different pathogenesis had encouraged us to target the RANK/RANKL pathway directly with this RANKL antibody as an individual translational therapeutic approach. Preliminary data of these four patients on biochemical bone turn-over markers in the course of a maximum of three treatment cycles have recently been published by our group [12]. To our knowledge, these new data about a two years’ experience are the first about denosumab treatment, side effects and efficacy determined by changes of the areal bone mineral density (aBMD) and vertebral morphometry in children with Osteogenesis imperfecta.

### Patient

The boys were born to three different consanguineous couples and presented with a severe phenotype of OI VI [4]. The clinical findings and clinical courses have been described in the former publication [12]. OI had been diagnosed clinically when the first fractures had occurred. Spine X-rays had revealed multiple vertebral fractures and deformities. A therapy with intravenous bisphosphonates had been started as described [12]. During bisphosphonate therapy treatment response was poor. All children were depending on a wheelchair. In these patients, we had identified the causal *SERPINF1* mutations and had discovered the genetic cause of OI VI in the course of a previous research project [6]. Additionally, Osteoprotegerin levels as an osteoclastogenesis inhibitory factor were analyzed in two of these patients and showed reduced values (3.0; 4.0 pmol/l [normal range 5.7 ± 0.42 pmol/l]).

Informed consent was obtained according to the Declaration of Helsinki and an individual translational therapeutic approach with the RANKL antibody denosumab (Prolia®, Amgen, Thousand Oaks) was started. Denosumab was injected subcutaneously with a dose of 1 mg per kg body weight. Oral supplementation with calcium (body weight ≥15 kg: 1000 mg per day; body weight ≤ 15 kg: 500 mg per day) was administered for 2 weeks after each injection. Additionally, vitamin D (body weight ≤ 30 kg: 500 international units per day; body weight ≥30 kg: 1000 international units per day) was prescribed in all patients because they were vitamin D depleted. Initially, treatment intervals were 12 weeks. These intervals were chosen according to the intervals used in adults [13]. After one year of treatment all patients were assigned to shorter intervals (minimum 10-week ) based on the recurrence of skeletal pain and on the increase of osteoclastic activity 8 weeks after injection measured by urinary deoxypyridinoline levels/creatinine (DPD) (data not shown).

## Methods

Dual-energy X-ray absorptiometry was performed at the lumbar spine (L2–L4) and for the total body (excluding the head) using a GE Lunar iDXA densitometer (GE Ultraschall GmbH, Solingen, Germany) and Encore software version 13.6. Areal BMD results were transformed to age-specific z-scores using reference data provided by the company [14].

We used urinary deoxypyridinoline/creatinine ratio (DPD/crea) to quantify and monitor the inhibition of bone resorption. Urinary concentrations of DPD were measured with a commercially available chemiluminescence assay (Pyrilinks®-D, Siemens Medical Solutions Diagnostics). The DPD/crea ratio was compared to age- and sex- matched reference data, as published by our laboratory [15]. Due to a change of the analytic procedures we had to analyze the later samples with High-Performance-Liquid-Chromatography with age matched reference data. Osteocalcin (Enzyme-immuno-Assay, reference ranges 10-100 ng/ml), Parathyroid hormone (Modular E-Modul, Roche Diagnostics, Germany, reference range 12–72 ng/l), 25-OH Vitamin D Modular E-Modul, Roche Diagnostics, Germany, reference range 30–70 μg/l) and total serum Calcium (Modular P-Modul, Roche Diagnostics, Germany, reference range 2.2-2.65 mmol/l) were measured in the serum by our university central laboratory.

Lateral x-rays of the spine were taken routinely. Spine morphometry was evaluated based on the semiquantitative score described by Koerber et al. assessing vertebral compression and deformity [16]. They were evaluated by the same radiologist. Additionally, calculation of the projected vertebral area of the lumbar vertebrae (L2-L4) was performed as described recently [17].

Mobility was assessed using the Gross Motor Function Measure (GMFM66) [18] and the Brief Assessment of Motor Function (BAMF) [19].

## Results

After two years of treatment all 4 patients were in a stable clinical condition. There was no treatment interruption based on unexpected side effects. Especially no signs of allergic reactions or clinical significant hypocalcemia have been reported. Patients 3 and 4 reported in the previous publication intermittently went back to their mother country for nearly one year. Therefore results of mobility and data of one year follow up were presented in detail only for patient 1 and 2.

### Clinical data

A synopsis of the clinical data and clinical course is given in Table 1. In three children, body length increased during denosumab treatment. One patient reached his final height before starting denosumab. Two patients suffered two fractures during the follow-up period due to mild traumata.

**Table 1:** **Clinical characteristics at the start and during denosumab treatment**

**Findings**	**Patient 1**	**Patient 2**	**Patient 3**	**Patient 4**
Mutation in *SERPINF1*	c.324_325dupCT	c.1132C > T	c.696C > G	c.696C > G
Duration of previous bisphosphonate treatment [years]	8.1	5.75	3.4	3.4
Age at start of denosumab therapy [years]	9.4	6.9	5.7	18.5
Time since first denosumab injection [years]	2.4	2.1	2.0	2.0
Number of denosumab treatment cycles [n]	12	10	8	8
Length [cm] (SD) at start	109 (−4.0)	102 (−3.55)	89 (−5.8)	120 (−9.9)
Length [cm] (SD) after two years	117 (−3.7)	107 (−4.3)	95 (−6.2)	120 (−9.9)
Weight [kg] / BMI [kg/m^2^] at start	19.7/16.6	14.7/14.1	10.8/13.6	51.4/35.7
Weight [kg] / BMI [kg/m^2^] after two years	28.2/20.6	17.5/15.3	11.0/12.2	54.0/37.5
BAMF mobility score at start	3	3	4	2
BAMF mobility score after one year	3	3	-	-
BAMF mobility score after two years	4	4	5	3
GMFM 66 score at start	32.31	34.84	-	-
GMFM 66 score after one year	31.78	40.20	-	-
GMFM 66 score after two years	34.37	40.67	-	-
Fractures under denosumab treatment [n]	2	2	0	0

*Abbreviations:* *BMI:* Body Mass Index, *BAMF:* Brief Assessment of Motor Function, *GMFM:* Gross Motor Function Measurement.

### Areal bone mineral density

Areal bone mineral density was assessed once a year.

**Figure 1:**
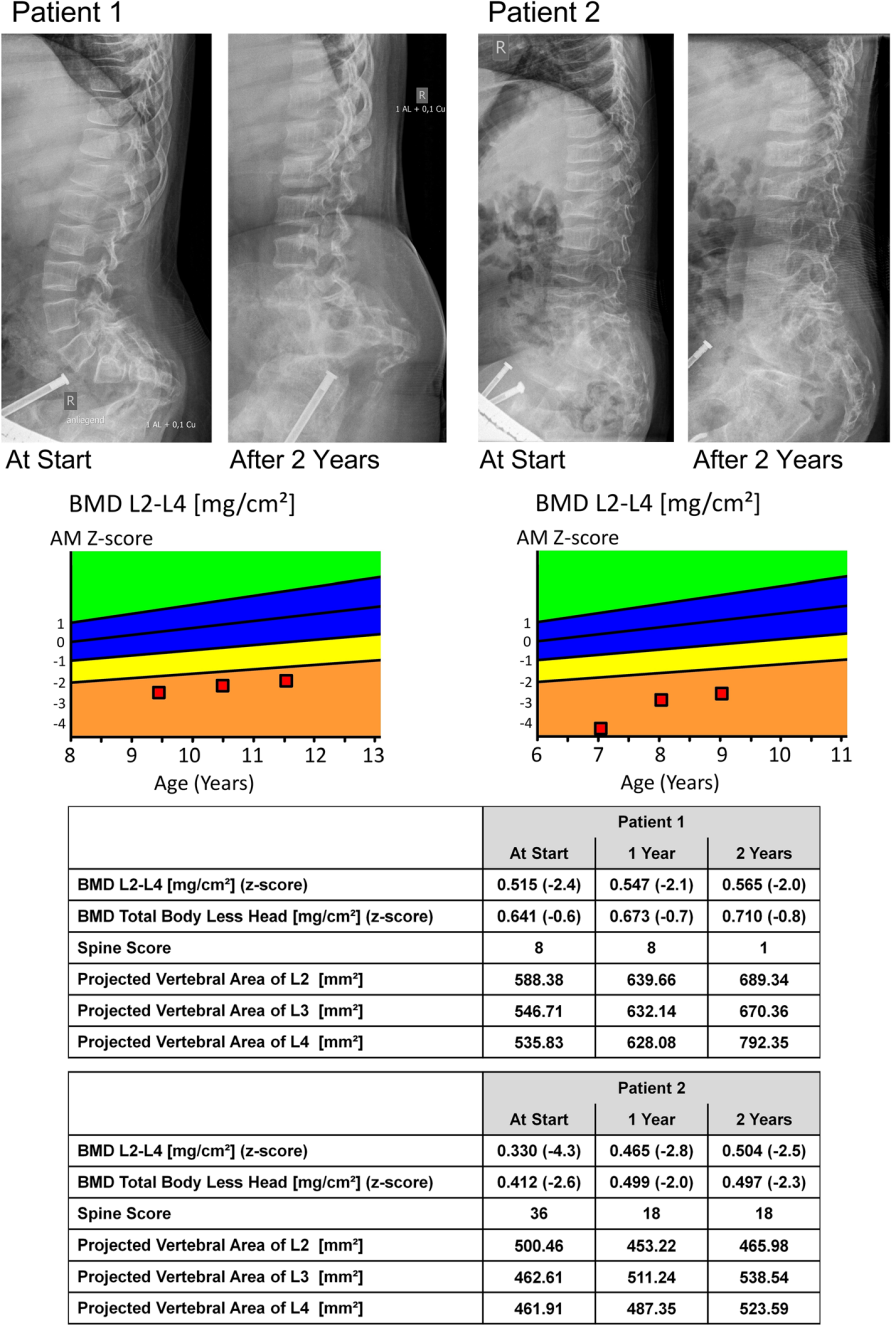
**Changes of areal bone mineral density, vertebral morphology and morphometry within the two years of treatment with denosumab in patient 1 and 2.** At the top, lumbar spine radiograms of patient 1 and 2 are presented at start and after 2 years of denosumab treatment showing a re-shaping of the vertebrae in both patients. In the middle, changes of lumbar areal bone mineral density at the starting point of the observation and after one and two years of therapy are presented graphically. At the bottom, data of the lumbar and total bone mineral density display an increase in both patients. Additionally, data of the spine score (vertebral compression and deformity) and projected vertebral area of lumbar vertebrae 2–4 demonstrate an improvement in both patients. Abbreviations: BMD L2-L4: areal bone mineral density of the lumbar vertebrae 2–4.

In all subjects, a continuous increase of aBMD was seen after one and two years of denosumab treatment. Age-matched z-scores and absolute values of patient 1 and 2 are presented graphically in Figure 1. In patient 3 and 4 aBMD (total body less head) changed from 0.373 g/cm^2^ (z-score −2.9) to 0.407 g/cm^2^ (z-score −2.9) and from 0.59 g/cm^2^ (z-score −2.8) to 0.633 g/cm^2^ (z-score −2.6), respectively at the only follow up visit after two years.

### Desoxypyridinoline level

A decrease of DPD excretion after denosumab injections into the reference range could be observed repeatedly. Data are presented exemplarily in Table 2 [15].

**Table 2:** **Changes of urinary deoxypyridinoline excretion during denosumab treatment**

	**Urinary deoxypyridinoline/creatinine ratio (age related normal range)**			
	**Patient 1**	**Patient 2**	**Patient 3**	**Patient 4**
**Days after injection**	**1** ^**st**^ **Cycle**	**1** ^**st**^ **Cycle**	**1** ^**st**^ **Cycle**	**1** ^**st**^ **Cycle**
**Day 1**	**44** (6.5-26.5 nM/mM)	**32** (6.5-26.5 nM/mM)	**82** (6.5-26.5 nM/mM)	**41** (0.0-17.8 nM/mM)
**Day 14**	**14** (6.5-26.5 nM/mM)	**25** (6.5-26.5 nM/mM)	**26** (6.5-26.5 nM/mM)	**20** (0.0-17.8 nM/mM)
**Day 28**	**25** (6.5-26.5 nM/mM)	**13** (6.5-26.5 nM/mM)	**25** (6.5-26.5 nM/mM)	**17** (0.0-17.8 nM/mM)
	**3** ^**rd**^ **Cycle**	**4** ^**th**^ **Cycle**	**8** ^**th**^ **Cycle**	**8** ^**th**^ **Cycle**
**Day 1**	**48** (6.5-26.5 nM/mM)	**78** (6.5-26.5 nM/mM)	**620** (110–450 μg/g)	**83** (10–50 μg/g)
**Day 14**	**10** (6.5-26.5 nM/mM)	**24** (6.5-26.5 nM/mM)	**133** (110–450 μg/g)	**50** (10–50 μg/g)
**Day 28**	**32** (6.5-26.5 nM/mM)	**19** (6.5-26.5 nM/mM)	431* (110–450 μg/g)	**23** (10–50 μg/g)
	**7** ^**th**^ **Cycle**	**10** ^**th**^ **Cycle**	-	-
**Day 1**	**42** (6.5-26.5 nM/mM)	**477** (110–450 μg/g)	-	-
**Day 14**	**28** (6.5-26.5 nM/mM)	**110** (110–450 μg/g)	-	-
**Day 28**	**19** (6.5-26.5 nM/mM)	**197** (110–450 μg/g)	-	-

Changes of urinary deoxypyridinoline levels (urinary deoxypyridinoline/creatinine ratio) presented exemplarily for different treatment cycles in all patients. Based on a change of the method reference ranges differed over the period and for clarity every reference range (as provided by the laboratories) are shown after every measured DPD level. The results of the first cycle and of previous bisphosphonate therapy have already been described in part [12].

*→ Value 3 days after elective surgery of right femur due to dislocation of intramedullary rod.

### Spine morphology

Changes of spine morphology of patient 1 and 2 during denosumab treatment in the lateral spine x-rays are presented in Figure 1. The vertebrae showed a re-shape phenomenon in both patients. A standardized assessment of the spine morphology revealed a stabilization and/or improvement in both children. The score levels are presented in Figure 1. Additionally, morphometric parameters are presented in Figure 1, showing an increase of the complete lumbar vertebral area of L2-L4.

### Mobility

The GMFM ranks showed stable results in both patients 1 and 2. GMFM 66 scores are presented in Table 1. The patients were not able to stand, walk, or jump, but at least mobility scores did not decrease further.

BAMF was additionally assessed every year and showed a slight increase after two years of denosumab treatment in all patients (Table 1).

### Safety

No severe side effects were reported by our 4 patients. All injections were well tolerated without any changes in vital signs. Serum calcium concentrations decreased repeatedly, and serum parathyroid hormone levels increased slightly (Table 3). There was no secondary hyperparathyreoidism observed over the whole period of two years. The slight hypocalcaemia after each injection could be compensated by oral calcium supplementation (lowest ionisized serum calcium level was 0.96 mmol/l, lowest total serum calcium level 1.97 mmol/l). Osteocalcin levels as a marker for bone formation stayed in the age related normal range in all patients (Table 3). No increased rate of infections was observed. Two patients suffered two fractures during the follow-up period due to mild trauma. The 3 patients which did not reach final height before start of treatment showed an increase of body length during the treatment period of 8 cm, 5 cm and 6 cm, respectively.

**Table 3:** **Bone metabolism markers in patient 1–4 in the last denosumab treatment cylce**

	**Patient 1**	**Patient 2**			**Patient 3**			**Patient 4**		
**Parameter/day after denosumab injection**	**Day 0**	**Day 0**	**14**	**28**	**Day 0**	**14**	**28**	**Day 0**	**14**	**28**
**Osteocalcin (age adjusted normal range) [ng/ml]**	24 (10.0-100.0)	45 (10.0-50.0)			40 (10.0-50.0)			21 (9.6-41.0)		
**Parathyroid hormone (12–72 ng/l)**	16	7	40	31	14	22	19	48	45	46
**Calcium (2.2-2.65 mmol/l)**	2.48	2.48	2.14	2.35	2.39	2.25	2.35	2.37	2.31	2.46
**25-OH Vitamin D (30–70 μg/l)**	19	15.4	23.2	26.7	8.2	8.8	4.3	12.2	10.8	8.1
**Desoxypyridinoline/creatinine (age adjusted normal range) [μg/g]**	600 (110–450)	477 (110–450)	110 (110–450)	197 (110-450	620 (110–450)	133 (110–450)	431* (110–450)	83 (10–50)	50 (10–50)	23 (10–50)

*→ Urin collected 3 days after elective surgery of right femur due to dislocation of intramedullary rod.

## Discussion

### Efficacy

Measurements of urinary DPD showed a suppression of bone resorption after denosumab application, and consequently, an increase of areal bone mineral density was observed in all patients. These are the major findings which underline efficacy of the denosumab treatment (Figure 1; Tables 2 and 3). In general, the clinical course of all patients improved as reported by the families. It is known that bisphosphonates are not as effective in children with OI VI as in children with other types of OI [5]. Therefore these results are interesting especially because both agents are “antiresorptive agents”. It might be argued that the large amount of osteoid which is characteristic for OI VI is responsible for a reduced efficacy of bisphosphonates. Bisphosphonates bind on the hydroxylapatites on the bone surface. Osteoid as the unmineralized compartment of the bone could not be bound by bisphosphonates and therefore the induction of osteoclastic apoptosis by bisphosphonates might not be as effective in OI VI as in other types of OI [5]. Denosumab as a RANKL antibody acts independently of the mineralization status of the bone, a fact that raises the possibility that this antiresorptive agent may be more effective in reducing osteoclast formation, maturation and bone resorption in patients with OI VI. Further trials are needed to compare the effect of a therapy with bisphosphonates and denosumab in children with OI.

A detailed analysis of the morphometric parameters of the radiographs taken from the lateral spine showed an improvement of the shape of the vertebrae which is essential for stability. This effect was shown by bisphosphonate treatment in children with typical OI (mutations in *COL1A1/A2*) but was not reported in OI VI [5,17].

The four patients with OI VI were severely affected. In this context, the clinical course during denosumab treatment was satisfying, as fracture rates decreased in all patients and mobility scores showed a slight increase. Additionally, patients reported reduced bone pain in the weeks following each denosumab injections, and a recurrence of bone pain at the end of each treatment cycle.

Based on our data no final conclusions about duration of treatment or frequency of injections can be drawn. It could be speculated that the treatment should be continued till the end of growth, similar to the currently used regime for bisphosphonates. Bone turnover is especially high during childhood and adolescents, and a strong effect of an antiresorptive treatment can be expected. A treatment until the end of puberty might also decrease the risk of a rebound effect after the end of therapy. However, these questions have to be answered in further trials.

### Safety

No severe side effects were observed. The observed mild side effects were those cited in the investigators brochure. The drop of serum calcium, a phenomenon which can be considered as an indicator for the decreased ability of osteoclasts to resorb bone and to release calcium, was easy to correct by oral supplementation and vitamin D supplementation. A decrease in calcium has also been reported in osteoporotic adults treated with denosumab [20] and is a common side effect of antiresorptive agents. This side effect was even used to treat hypercalcemia in 2 children after stem cell transplantation in osteopetrosis [21]. Intravenous calcium substitution as it was described in a child with juvenile Paget disease was not needed [22]. At the end of each treatment cycle no hypercalcemia as it was reported for other patients [22,23] was observed in our patients. The previous bisphosphonate treatment may have attenuated this rebound effect. However, the patients reported in the literature also received bisphosphonates prior to denosumab. Another reason for the absence of hypercalcemia in our patients might be the supplementation regime for calcium, which we administered for only 2 weeks after the denosumab injections, compared to a continuous treatment in the patient with paget disease. Additionally, the differing pathophysiology in patients with OI type VI may have influenced the effect of denosumab on serum calcium levels.

In our patients no increase of fractures, bone pain and infections has been reported by the patients and their parents. No growth arrest was observed. The observed height velocity was reduced in comparison with healthy children but was not reduced in comparison to the prior period under bisphosphonate treatment.

After two years of application, the benefits seem to overweigh the risks of treatment.

In the long term, denosumab might offer an advantage in different disorders with increased bone resorption because it completely degrades within 3–6 months after an injection of 1 mg/kg body weight [24]. Bisphosphonates are stored in the bone persistently, a fact which has led to an ongoing debate about their long-term safety for children [25].

### Limitations

Limitations of this brief report are that only two-year data for four patients with OI VI were available, a consequence of the fact that OI VI is extremely rare, with only a few patients currently known in Germany. This small sample size limits the conclusions that can be drawn from our data. No controlled setting is available to compare the presented effects with bisphosphonate efficacy. Recently, several case reports about the short-time use of denosumab in children with different conditions became available, but there are no long-term data on the use in children [21-23,26].

Even though this report underlines that denosumab is well tolerated, the treatment period of two years is still too short to predict that it will be safe also in the long-term course, as e.g. allergic reactions or antibodies targeting denosumab may require time to develop. The patients have to be monitored longer in order to evaluate the risk-benefit ratio more precisely.

In conclusion*,* medium-term treatment with denosumab seems to be safe. No severe side effects were seen, and no growth arrest was observed in the presented patients. Efficacy seems to be superior to earlier bisphosphonate treatment. Treatment with denosumab led to an increase of areal bone mineral density and mobility, as well as to a marked and reversible suppression of bone resorption. Further controlled trials are needed to assess the long-term effects in comparison to bisphosphonate treatment prospectively. Last but not least this report is a striking example of the fact that a precise molecular diagnosis can have immediate implications for the therapeutic regime, and that the period of time required to transfer new scientific insights from “bench to bedside" can sometimes be very short – in this case, less than a year.

## Informed consent

All procedures followed were in accordance with the ethical standards of the responsible committee on human experimentation (institutional and national) and with the Helsinki Declaration of 1975, as revised in 2000. Informed consent was obtained from all patients/parents for being included in the study.
